# Conceptualization of Rice with Low Glycaemic Index: Perspectives from the Major European Consumers

**DOI:** 10.3390/foods11142172

**Published:** 2022-07-21

**Authors:** Diva Cabral, Susana Caldas Fonseca, Ana Pinto Moura, Jorge C. Oliveira, Luís Miguel Cunha

**Affiliations:** 1 GreenUPorto—Sustainable Agrifood Production Research Centre/Inov4Agro, Rua da Agrária 747, 4485-646 Vila do Conde, Portugal; diva.cabral@fc.up.pt (D.C.); susana.fonseca@fc.up.pt (S.C.F.); apmoura@uab.pt (A.P.M.); 2 DGAOT, Faculty of Sciences, University of Porto, 4485-646 Vila do Conde, Portugal; 3 DCeT, Universidade Aberta, 4200-055 Porto, Portugal; 4 School of Engineering and Architecture, University College Cork, College Road, T12 YN60 Cork, Ireland; j.oliveira@ucc.ie

**Keywords:** consumers’ perceptions, consumption, free word association, glycaemic index, Portugal, rice

## Abstract

Rice and cereal consumption has become a concern for consumers due to usually high glycaemic indexes (GI), which is a critical issue for a balanced and healthy diet. Therefore, the development of new products with low GI is an important target of the industry, particularly in countries with high consumption. This study assesses consumers’ perceptions about “rice” and “rice with low GI” and evaluates the effect of consumers’ rice consumption profiles through the application of a free word association technique in a structured self-administered electronic questionnaire with 256 Portuguese consumers (the European market with the highest per capita consumption of rice by far). The frequency of rice consumption was evaluated, and the consumption profile was determined through a hierarchical cluster analysis, with 9% identified as daily consumers. The response words were categorized by the triangulation technique, and the association between the word categories and dimensions, sociodemographic characteristics, and consumption profile were determined. Respondents most frequently associated “rice” with rice dishes, its sensory attributes, and nutrition, highlighting the satisfaction of nutritional and hedonic needs. Consumers revealed positive expectations in relation to the functionality of “rice with low GI”. The consumers’ rice consumption profiles, sex, age, and educational levels influenced their perception towards “rice“ and “rice with low GI”. This study provides important insights for the industry to develop a consumer-oriented, low GI rice product.

## 1. Introduction

Rice (*Oryza sativa* L.) is the staple food of most of humankind, with a world per capita consumption of about 80.6 kg/year. The European average value of consumption is more modest, at just 6.7 kg/capita/year, registered between 2015 and 2019 [1]. Within this region, Portugal has the highest consumption, with an average of about 16.1 kg/year. Rice plays a relevant role in Portuguese cuisine, being used as a main course, as a side dish, and even as a dessert [2]. It accounts for 5.2% of the total energy intake, higher than its most direct carbohydrate competitors, namely, potato and pasta, which contribute to 4.6% and 3.0%, respectively [3]. In Portugal, there are two main commercial rice types, namely, *Carolino*, which is a long grain Japonica variety with milled kernels over 6mm in length, a length/width ratio between 2 to 3, and an amylose content below 22% (expressed in dry matter); and *Agulha*, which is a long grain Indica variety with milled kernels also over 6 mm length, but with a length/width ratio ≥ 3 and an amylose content above 25%, according to the Portuguese legislation DL 157/2017. *Carolino* rice is produced from different cultivars, *Ariete* being the most common, and is traditionally used to make rice dishes with a creamy texture. It is often cooked in a traditional way that uses abundant water, and the high amylopectin content results in the rice absorbing the broth it is cooked with to obtain rich flavours, derived from the ingredients being cooked together, such as tomato, vegetables, pulses, meat, fish, or shellfish. As it absorbs much more water than other types of cooking, it seems to “produce more food” by just using water, and therefore this way of cooking is traditionally known as “*Malandro*”, which means “cheater”.

Rice may be served as a side dish or as a main course, depending on the major ingredient added to the rice. Although *Agulha* and *Carolino* are the most consumed types of rice, other white rice types such as *Arborio* and *Carnaroli* (the Japonica varieties used for *Risotto*), and the aromatics Basmati and Jasmine are also becoming increasingly popular [4]. Rice can be consumed just de-husked, still with its bran layer, which is most typically brown rice, although other bran colours can be found, namely red, black, and purple. This is becoming more common due to the health benefits of bran. However, the vast majority of the consumption of traditional cuisine is with milled rice, all of it white once the bran is removed (by abrasion,) and thus white rice and milled rice are synonymous.

Several nutritional studies have shown that the excessive consumption of white rice is associated with an increased risk of non-communicable diseases (NCDs), namely diabetes, hypertension, obesity, and cardiovascular diseases [5,6,7,8,9,10]. The amount and nature of its refined carbohydrates (CHO) turns white rice into a food with a high glycaemic index (GI) [11]. The GI is an indicator of the health quality of the carbohydrates (CHO) present in foods based on how quickly blood glucose levels rise following digestion.

Based on the GI (considering glucose as a standard food), foods are considered as presenting high GI (GI ≥ 70), intermediate GI (55 < GI < 70), or low GI (GI ≤ 55) [12]. Foods with high GI release glucose rapidly into the blood, and foods with a low GI tend to release glucose slowly and steadily. Thus, foods with a low GI are likely to improve blood glucose and lipid control as well as promote insulin sensitivity and thus are beneficial dietary treatments for diabetic patients [13]. The GI of cooked rice ranges from 37 to 151, depending on variety, processing, and recipe factors [14,15,16,17]. Among milled (white) rice, the rice with the lowest GI are Basmati, *Doogara*, and some hybrid rice [18,19]. Rice from the Japonica subspecies (local rice) has the highest GI, with Portuguese varieties *Ronaldo* and *Ariete* having GIs of 89 and 151, respectively [16]. Brown (whole grain) rice and parboiled rice (rice pre-treated with steam prior to milling) tend to have the lowest GI compared to their polished forms [18,20].

This is particularly relevant, as NCDs are one of the most serious public health concerns in Portugal [21]. In 2015, cardiovascular diseases represented 29.7% of total deaths [22], with diabetes affecting approximately 10% of the Portuguese population, and the prevalence of high blood pressure being approximately 36% [23]. Obesity affects more than 20% of Portuguese adults, together with overweightness, which affects more than 50% of the population [3]. Prevalence of childhood overweightness (including obesity) is also high, estimated at 29.6% in 2019 [24]. In the same way, over the last decade, consumers consider in general that CHO negatively affects health, namely, as a cause of weight gain [25]. This is particularly relevant for consumers, as studies conducted in Western societies have shown that health is operating as an important individual food choice criterion [26]. In fact, more and more consumers believe that foods contribute directly to their health, and eating healthy products may prevent nutrition-related diseases and improve physical and mental wellbeing [27].

It is therefore very important for the rice industry to understand better how consumers perceive the connection between diet-related health and the consumption of rice. To evaluate consumer perceptions, researchers recur to direct methods, such as questionnaires, focus groups, or interviews, and to indirect methods such as free word association or observational research [28], the latter methodology having the advantage of being better able to grasp consumer intuitive and automatic behaviours [29].

Free word association (FWA) is a projective technique used to achieve associative memory and has gaining popularity in food consumption research, as it encourages respondents to project their underlying motivations, beliefs, attitudes, and/or feelings regarding a specific food [30,31,32]. It consists of presenting a series of words (stimuli words or target words) to a respondent, encouraging an immediate response by associating them with the first words that come to mind. Word association tasks are simple and easy to use and offer powerful insights into the concepts being tested [32]. This cognitive task, which involves the conceptualization of the stimulus, allows for the assessment of the knowledge, the degree of familiarity, and interest in relation to the topic under study. The first ideas to come to mind or the most recurrent might be the most relevant, for example, for purchasing decisions [33].

The FWA technique was also used to explore consumers’ perceptions concerning wellbeing in a food-related context [27] to investigate default attitudes toward food [34] and to assess the perceived cross-cultural values of consumption in a triadic approach [35]. The typology of consumption values is derived in terms of the product’s/service’s ability to have an end in and of itself or to serve as a means to a specific end, which includes utilitarian, symbolic, experiential, and aesthetic values [36]. For food consumption, a shorter triadic approach without aesthetic value proved to be interesting [35,37,38]. Within these, the utilitarian values are determined as a function of the food’s capacity to reach the final objective due to a specific characteristic of that food; the symbolic values are determined from intangible concepts, such as cultural and ideological, or other concepts related to the belief itself [39]. The achievement of an end is also latent to these values; however, it is not dependent on the physical attributes of the product. Finally, the experiential values are related to sensory stimulation and affective and emotional reactions associated with consumption. Some research has been done on the perceived values of consumption, where foods have been characterized according to their dominant values as well, showing that the relative importance of consumption values is culturally dependent [34,35,38,40,41]. For example, the consumption of rice by Asian consumers is dominated by utilitarian values, while symbolic values predominate for French consumers [35].

The aim of this study is to evaluate how rice with low GI is perceived and understood by Portuguese consumers compared to the more common white rice with high GI values. This is particularly relevant for the food industry, which intends to develop new products or improve existing products to meet consumers’ demands.

## 2. Materials and Methods

### 2.1. Participants

Over 250 participants living in the Great Oporto area were recruited considering the following inclusion criteria: (i) consuming rice at least once a month; (ii) above 18 years old, and (iii) willing to participate in the study. Despite being a convenience sample, it focused on a specific target group (rice consumers), and as such it may be considered as a reliable sample, commonly used in qualitative research [42].

The respondents were recruited by a sensory analysis and market research company from the North of Portugal named Sense Test. The company ensures the protection and confidentiality of data through the authorization 2063/2009 of the National Data Protection Commission and following EU Regulation 2016/679, as well as a longstanding internal code of conduct. The recruitment and scheduling of the inquiry were performed following a telephone-based invitation where only general information about the survey was provided.

All participants followed an informed consent procedure before answering the questionnaire.

### 2.2. Data Collection

In order to obtain consumers’ intuitive and spontaneous ideas, an FWA approach was applied. Each participant was asked to write the first three words that came to mind when they read the stimulus word “rice” (*arroz,* in Portuguese) on the screen, showing three blanks reserved for filling in, followed with the same question to the stimulus “rice with low glycaemic index” (*arroz com baixo índice glicémico,* in Portuguese). At the time of the questionnaire application, each respondent was taken to a quiet room and filled out the questionnaire that was presented on a computer screen. Data collection was done through a structured self-reported electronic questionnaire, using Lime Survey, and further included the evaluation of rice consumption patterns. The frequency of consumption of overall rice and of each different type of rice was asked, using close-ended questions, following a typical food frequency questionnaire [43]: (1) 1 to 3 times per month; (2) once a week; (3) 2 to 4 times a week; (4) 5 to 6 times a week; (5) once a day; and (6) 2 or more times per day. All types of rice in the Portuguese market were included in the questionnaire options, namely, Arborio (*Risotto*), *Agulha*, Basmati, *Carolino*, brown, wild, Jasmine (Thai Jasmin), and parboiled. Although wild rice (*Zizania aquatica* and *Z. palustris*) is not the same species as the others (*Oryza sativa* L.), it was included in the study as both are marketed as rice with no clear distinction between species. Additionally, sociodemographic data were also collected. The questionnaire also included the collection of age, sex, education level, and monthly household income. Data were collected over a two-month period, from February to March 2018.

### 2.3. Data Analysis

Data analysis of the FWA results was initiated with a spell check and correction of all response words. Next, the categorization of the response words was done by three experienced researchers using the triangulation technique [44]. This technique is widely used in content analysis to reduce the subjectivity related to this type of analysis [45]. The researchers, both individually and independently, grouped the words into exclusive categories and then into dimensions that were more comprehensive, considering the semantic and lexical relations according to the Portuguese language dictionary. The following assessment criteria were taken to each response word: (i) did not include words that elicited multiple interpretations in the context of the stimulus word (ambiguous words); (ii) maintained the same form for words that allow plural or singular and female or male; and (iii) did not include words that had no type of connection to the stimulus word, or made no sense; however, when the ambiguous or presumptively out-of-context words were in sounding numbers, they were grouped for later analysis and joint decision-making.

After the individual categorization of the response words by each researcher, a consensus categorization was achieved. This analysis was done in the native Portuguese language and only after was it translated into English using the rules established by Anderson and Brislin [46].

The frequency of each category and dimension was determined by word, counting the number of response words, and summing those in the same category and dimension; and by participant, counting the number of respondents that mentioned those categories or dimensions. The word frequencies were counted without considering if the words were evoked by the same respondent or not [47,48,49]. To avoid losing valuable information about the perception of cognitive associations of smaller groups, all the categories referred by at least 5% of the participants were considered for analysis.

In addition to grouping the words according to their semantic meaning, words were also grouped according to the perceived value into utilitarian, experiential, and symbolic. There are a variety of definitions and conceptualizations of value that depend on both the context of the study and the methodology and measurement techniques, as well as the theoretical background of the study (such as economic theory and cognitive psychology or consumer behaviour psychology) [40,50,51,52,53]. This has followed the classification consolidated by Lanseng [36] resulting from the analysis of the relevant literature that reflects on this topic.

To draw a rice consumption profile, a hierarchical cluster analysis was applied over the consumption frequency data for the different rice types using the Ward method for agglomeration and the square Euclidean distance as the similarity measure. The nonparametric Kruskal–Wallis test followed by pairwise comparison was also used to compare the frequency of consumption by type of rice between clusters.

A chi-square test of independence was used to evaluate if there is a significant relationship between FWA dimensions, rice consumption profile, and the sociodemographic variables (age group, sex, and education level), following a 95% confidence level. A chi-square test per cell was used to identify the source of the global chi-square variation through the adjusted standardized value [54,55,56]. To compare the consumption values associated with each stimulus word, the same analysis procedure was followed.

Statistical data analysis was processed with XL-STAT^®^, v. 2020.5.1 (Addinsoft, New York, NY, USA).

## 3. Results

### 3.1. Characterization of Participants

The questionnaire was applied to 256 Portuguese respondents aged between 18 and 73 years. Table 1 shows the sociodemographic characteristics of the participants in terms of age, sex, education level, and monthly household income.

### 3.2. Conceptualization of the “Rice” and “Rice with Low Glycaemic Index” Stimuli

**Figure 1:**
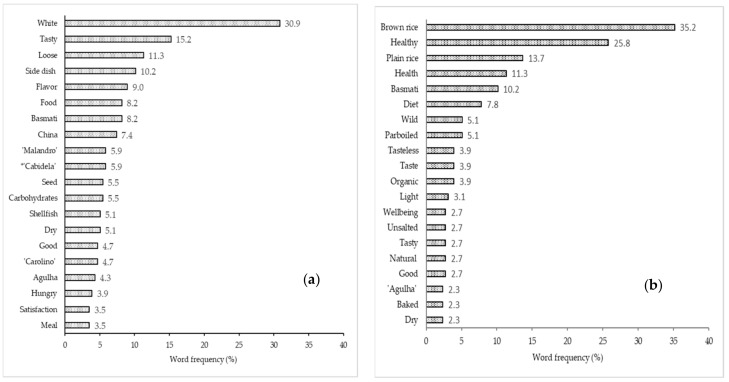
Word frequency of the twenty most evoked words for the stimulus: (**a**) “rice” and (**b**) “rice with low glycaemic index”. * *Cabidela* rice is a traditional *Malandro* rice dish of the gastronomy of the Northern region of Portugal made with *Carolino* rice, poultry, and chicken blood with vinegar, where the offal is also incorporated, resulting in a creamy/saucy rice with meat served as a main course.

A total of 1498 different terms was generated after consumers were invited to write the first three words that came to mind when thinking about “rice” and “rice with low GI”. Figure 1 shows the frequency of the 20 most evoked words in the FWA task for the stimulus (a) “rice”, and (b) “rice with low GI”. While ‘white’ and ‘tasty’ were the most frequent words for the “rice” stimulus, they were ‘brown rice’ and ‘healthy’ for the “rice with low GI” stimulus (Figure 1).

#### 3.2.1. “Rice” Stimulus

The FWA technique applied to the “rice” stimulus gave rise to 768 written words (all the respondents evoked the three words) that corresponded to 188 different response words. The four most recurrent response words were ‘white’ (30.9%), ‘tasty’ (15.2%), ‘loose’ (11.3%), and ‘side dish’ (10.2%) (Figure 1a).

**Figure 2:**
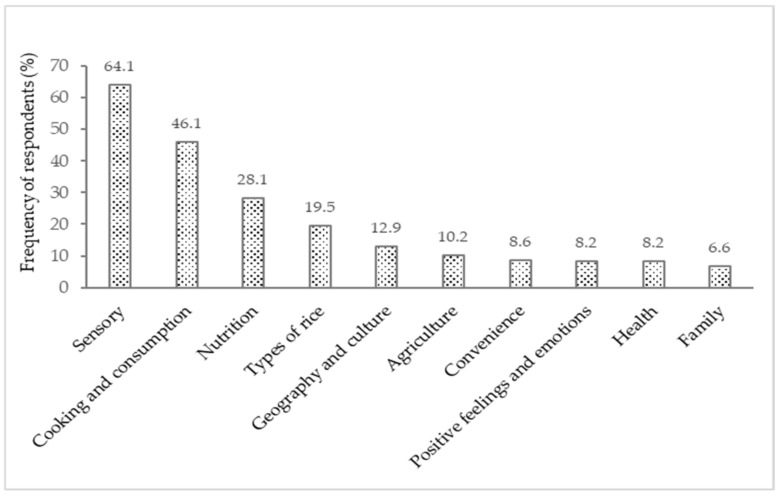
Frequency of respondents (%) describing words according to the dimensions for the “rice” stimulus.

The response words were grouped into 18 categories and 10 dimensions. Figure 2 presents the frequency of respondents according to each dimension for the “rice” stimulus, and Table 2 shows the frequencies of words and respondents according to each category with examples of the elicited words. The category ‘distribution and price’ (e.g., words: price, hypermarket, cheap, affordable, packaging, brand) was mentioned below the established cut-off point, and therefore was not considered for further analysis.

More than half of the respondents (64.1%) mentioned the ‘sensory’ dimension, corresponding to 34.2% of words, in which the main components of the rice quality assessment were elicited, related to shape, colour, integrity, results, cooking, and grain processing. ‘Appearance’ (16.3% of words) was the largest category of the “rice” stimulus, and the main sensory attribute cited far more often than the other attributes such as ‘flavour’ (9.8% of words) and ‘texture’ (4.2% of words).

The second most expressive dimension was ‘cooking and consumption’ with 24.3% of words and 46.1% of respondents, which refers to methods of preparing and ways of consuming rice. This dimension was comprised by ‘specific foods’ (shellfish, tuna, beans, tomato, bacon, chicken, duck, etc.) that are commonly used as ingredients in culinary preparation (8.0% of words), ‘rice side dish’ (7.6% of words), and ‘rice main course’ (5.8% of words), as well as ‘culinary practices’ (2.9% of words). In the category ‘culinary practices’, some cooking methods were evoked, such as ‘braise’ and ‘*estrugido*’, which are common ways of cooking rice. ‘*Estrugido*’ (Portuguese word) is a specific term for braising rice, which is frying garlic and onion in olive oil (may include other condiments) and gaining colour without burning, where other ingredients of the dish to be cooked are later added.

The ‘nutrition’ dimension contained the ‘staple/sustenance’ and ‘nutritional aspects’ categories, revealing the functional dimension of rice. Due to the importance of rice in the Portuguese eating habits, associations with the basic nutritional characteristics (‘staple/sustenance’ category) were already expected. In this category, words such as ‘basic’, ‘essential’, ‘food’, ‘eating’, ‘to eat’, and ‘staple’ were mentioned. The ‘types of rice’ dimension was mentioned by 19.5% of respondents who mentioned various types of rice such as Basmati, *Carolino*, *Agulha*, *Risotto*, brown, parboiled, wild, and Jasmine. It is noted that consumers will freely describe as ‘types of rice’ a mix of actual names of varieties, commercial names, and forms of cooking. For instance, *risotto* is an Italian way of cooking rice, not a variety or type of rice. Actually, Portuguese restaurants have been known to cook *risottos* using *Carolino* rice; there is nothing wrong with that because Portuguese consumers do not actually appreciate the *al dente* texture that would require varieties like Arborio or Carnaroli to be used. This mix-and-match of names is what consumers read in the packages that they buy, and thus these loose designations were maintained in the text.

‘Positive feelings and emotions’ include terms such as ‘comfort’, ‘enjoyment’, ‘fun’, ‘joy’, ‘passion’, ‘pleasure’, ‘spectacular’, and ‘success’ (3.4% of words and 8.2% of respondents), which are words that express positive feelings and affective aspects of consumption.

The ‘geography and culture’ dimension brought together names of countries and terms related to specific culture and habits. ‘Agriculture’, ‘convenience’, ‘health’, and ‘family’ were the last dimensions associated with rice. ‘Convenience’ was positively referred to, associating words such as ‘easy’, ‘fast’, ‘practical’, and ‘versatile’. The versatility and practicality are related to the wide culinary applicability of rice, as well as the existing varieties.

#### 3.2.2. “Rice with Low Glycaemic Index” Stimulus

This stimulus had a total of 730 written words, with 265 different response words, with ‘brown rice’ (35.2%) and ‘healthy/health’ (25.8% + 11.3%) being the most evoked words (Figure 1b). Thirty-nine missing values (non-responses) were obtained, corresponding to 8.6% of respondents. Among the twenty most frequent, other words were also evoked, such as ‘plain rice’ (13.7%), ‘Basmati’ (10.2%), ‘diet’ (7.8%), ‘organic’ (3.9%), ‘natural’ (2.7%), as well as some sensory descriptors, namely, ‘flavour’, ‘tasteless’, and ‘dry’.

**Figure 3:**
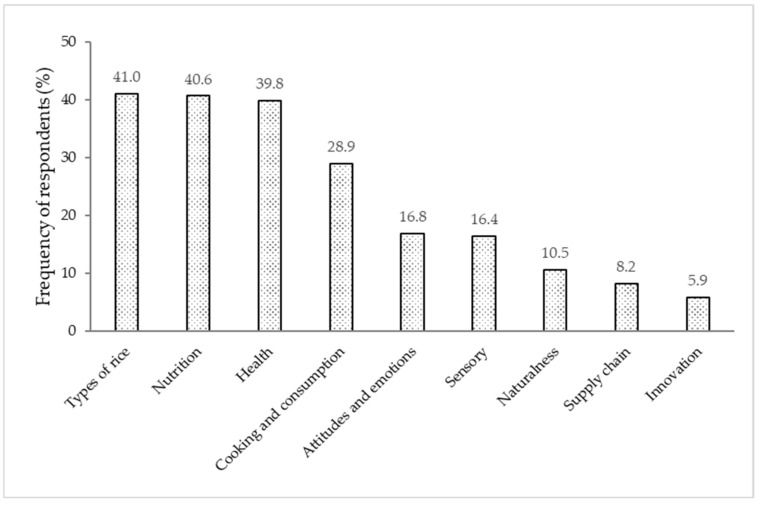
Frequency of respondents (%) describing words according to the dimensions for the “rice with low glycaemic index” stimulus.

The “rice with low GI” stimulus gave rise to 19 categories; of these, the following ones were not considered due the low reference frequencies (cut-off point: minimum 5% of respondents): ‘other types of rice’ (*Agulha*, *Carolino*, Jasmine), ‘appearance’, and ‘texture’, decreasing to a total of 16 categories. These categories were grouped into nine dimensions presented in Figure 3. Table 3 shows the frequency of words and respondents according to each category with examples of the elicited words.

The most referred dimension was ‘types of rice’ (19.4% of words and 41.0% of respondents). The ‘nutrition’ dimension (17.0% of words and 40.6% of respondents) was composed of ‘nutritional aspects’, where the terms related to the nutritional characterization of the stimulus and main nutrients of rice were grouped, while the category ‘diet patterns’ consisted of terms related to the usual food intake and terms relating to food restriction or reduction. This dimension also included the ‘low GI disconnects’ category, which gathered incoherent nutritional aspects in the context of this stimulus, such as ‘gluten free’, ‘more vitamins’, ‘with more vitamins’, and ‘more phosphorus’. There was also the association of this stimulus to ‘unsalted’ and ‘non-fat’, where about 11.7% of respondents made this kind of association.

The ‘health’ dimension (20.5% of words and 39.8% of respondents) was composed of the categories ‘physiological’ and ‘health benefits’. The ‘physiological’ category grouped terms related to the organic functions or vital processes of the human organism. The ‘health benefits’ category includes terms associated with achievements driven by healthy eating and some specifically by consumption of low GI foods.

The ‘rice dishes’, ‘specific foods’, and ‘culinary practices’ categories made up the ‘cooking and consumption’ dimension. In ‘culinary practices’, healthier cooking methods were mentioned, such as ‘grilling’, ‘boiling’, and ‘steaming’. In the ‘naturalness’ dimension, the concern was with both production and processing, mentioning terms such as ‘organic’, ‘additive-free’, ‘preservative-free’, and ‘unprocessed’.

In the ‘sensory’ dimension, some limiting attributes of liking were evoked, such as ‘bitter’, ‘tasteless’, ‘little taste’, ‘insipid’, and ‘no-taste’. However, there was also mention of attributes indicating positive attitudes and feelings such as ‘satisfaction’, ‘happiness’, ‘joy’, and ‘good mood’ grouped in the ‘attitudes and emotions’ dimension. Related to this dimension, terms associated with negative feeling (e.g., ‘doubt’, ‘dissatisfied’, ‘uncertainty’, ‘unfamiliarity’) also appeared. The ‘positive attitudes and emotions’ category highlighted a positive perception of rice with low GI, evoking words such ‘suitable’, ‘interesting’, ‘advisable’, ‘ideal’, ‘better’, ‘essential’, and ‘special’.

The last dimensions were ‘supply chain’, which cited some rice products (rice drink, tufted rice, rice flour, instant rice, dehydrated rice), and ‘innovation’, which made perfect sense for this concept, since the stimulus incited something new for the respondents. These terms allude to innovation and rice products that can be clues to the intended low GI products. These least frequent dimensions (‘supply chain’, ‘innovation’) also referred to the way of accessing “rice with a low glycaemic index”.

#### 3.2.3. Consumption Values for “Rice” and “Rice with Low Glycaemic Index” Stimuli

**Figure 4:**
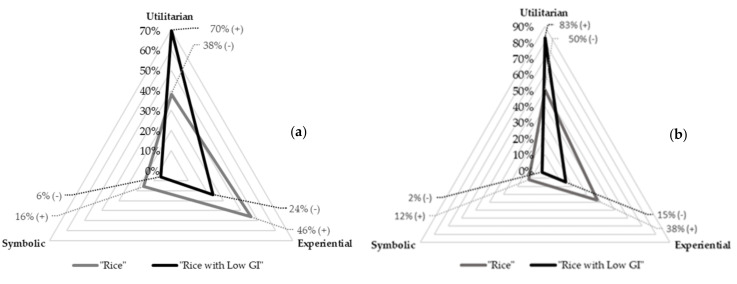
Frequency of (**a**) respondents and (**b**) words associated with each consumption values (utilitarian, experiential, symbolic) and stimuli (“rice” and “rice with low GI”). Effect of the chi-square per cell: (+) or (−) indicate that the observed value is significantly (*p* < 0.001 for all comparisons) higher or lower than the expected theoretical value.

When addressing the consumption values associated with each of the elicited words, in accordance with the stimuli concepts “rice” and “rice with low glycaemic index”, clear differences emerged, both based on the frequencies of words and participants. These results are shown in Figure 4.

The response words evoked by the respondents from the “rice” stimulus were mostly of an experiential nature (46%), followed by utilitarian (38%), and finally the symbolic values, with 16% of respondents. The most evoked dimension (sensory) carried experiential value, but most dimensions followed predominantly utilitarian values.

In the association task with “rice with low GI”, most response words represented utilitarian consumption values (70% of respondents), demonstrating the functionality of rice with low GI. There were 24% of respondents who expressed words with experiential values. These words were grouped in the ‘attitudes and emotions’ and ‘sensory’ dimensions. Symbolic values were the least mentioned with only 6% of respondents.

Through the chi-square test, it was verified that there were significantly more associations of experiential and symbolic values to the stimulus “rice”, while for the stimulus “rice with low GI”, the respondents made significantly more utilitarian associations.

### 3.3. Rice Consumption Profiles

To better understand the respondents’ rice consumption profiles, a hierarchical cluster analysis was performed based on the consumption frequency of each type of rice. Four clusters were obtained and labelled as follows (Table 4):

- Specialities Cluster—consumers which stood out for their specialty rice types (Basmati, Jasmine, Risotto, brown, parboiled) consumption and with the lowest frequency of rice consumption in general (overall rice consumption: 3.6 times/week);
- Local Cluster—most frequent consumers of Carolino rice (a Portuguese rice type) and Agulha rice (the most consumed rice type), and those who consume fewer specialty rice types (overall rice consumption: 4.2 times/week);
- Daily Cluster—group with highest weekly consumption for all types of rice, depicting a daily consumption of rice (overall rice consumption: 6.8 times/week);
- Agulha Cluster—most frequent consumers of Agulha rice (overall rice consumption: 4.5 times/week).

Table 5 shows the relationship between the rice consumption profiles (four clusters) and the respondents’ sociodemographic characterization variables (age group, sex, education level, and monthly household income). There were significantly more male respondents in the Local cluster (consumption of the most common rice types and lower consumption of specialities) than female, while for the Specialities and Daily clusters, the opposite was verified. There were significantly more respondents with higher education in the Specialities cluster, and more respondents without higher education in the Local cluster.

### 3.4. Evaluation of the Relationship between the Free Word Association Categories, Dimensions, and Values, the Rice Consumption Profiles, and the Sociodemographic Variables

To perceive the relationships between variables emerging from free association (categories, dimensions, and values), consumption profile, and sociodemographic variables (age group, sex, educational level, and monthly household income), the chi-square independence test was performed for both stimuli with a 0.05 significance level.

#### 3.4.1. “Rice” Stimulus

Table 6 presents the results of the associations considering the rice stimulus. Results showed that male respondents made significantly more associations to the ‘specific foods’ (which are normally ingredients for the preparation of rice dishes or to complement the rice meal) and ‘geography and culture’, while female respondents mentioned significantly more ‘types of rice’, ‘sensory’, and ‘family’. The oldest respondents made significantly more associations to ‘nutrition’, ‘positive feelings and emotions’, ‘agriculture’, ‘convenience’, and ‘health’, and the younger, in turn, made significantly more associations with ‘appearance’. The middle age group associated more positively with ‘specific foods’.

Respondents with higher education seemed much more often to consider the ‘rice as side dish’ and ‘type of rice’ categories in their associations than individuals without higher education. Those without higher education more frequently associated the “rice” stimulus to ‘flavour’, ‘positive feelings and emotions’, ‘convenience’, and ‘health’.

As for the rice consumption profile, it was found that the Specialities cluster mentioned significantly more ‘types of rice’ and ‘agriculture’. The high frequency rice consumption cluster made significantly more associations to the ‘convenience’ category. The cluster with the highest *Agulha* rice consumption was the one that made most mention of the words related to mood (‘positive feelings and emotions’), which were significantly less mentioned by the Local cluster.

The female respondents and *Agulha* clusters mentioned significantly more response words of experiential value. Symbolic values, which were the least associated with the concept (16%, Figure 4), were significantly more often mentioned by the Specialities cluster.

#### 3.4.2. “Rice with Low Glycaemic Index” Stimulus

The associations considering the “rice with low GI” stimulus are presented in Table 7. ‘Types of rice’ was mentioned significantly more by female and higher education respondents. In the nutritional dimension, the youngest cited more terms related to the ‘nutritional aspect’. ‘Low GI disconnects’ was evoked significantly more by respondents without higher education. ‘Cooking and consumption’ was evoked significantly more by the middle-aged group ([35; 55[) and by respondents without higher education. In the dimension ‘attitudes and emotions’, male respondents were the ones who most evoked ‘negative feelings and emotions’, while ‘positive attitudes’ was significantly more often mentioned by the older ones. Older respondents also evoked significantly fewer sensory terms and evoked significantly more terms from the ‘naturalness’ dimension. The terms related to ‘innovation’ were significantly more often mentioned by male, younger, and higher education individuals.

Rice dishes were mentioned more by Local consumers, while the Specialities consumers mentioned significantly more ‘types of rice’ and significantly less ‘health’ and ‘sensory’ dimensions.

For consumption values, significant differences were found between Specialities and Local consumers. It was found that the Specialities consumers mentioned significantly more response words with utilitarian values, while the Local cluster mentioned significantly more experiential values.

## 4. Discussion

The findings of this research emphasize the long experience of Portuguese consumers in the context of rice in their meals. In the FWA task, all respondents filled in the three reserved spaces for the “rice” stimulus. For the “rice with low GI” stimulus, 10% of the respondents left at least one blank, and the level of response divergence was greater, resulting in a greater number of different words, indicating that our respondents were less familiar with this concept, as the familiarity of the participants with the stimulus determines how they will process information to answer questions [57,58].

For the “rice” stimulus, the most frequent words evoked by our participants were ‘white’, ‘taste’, and ‘loose’. This revels that the ‘sensory’ dimension represents the main rice consumer association, as the higher the frequency of elicitations the greater the salience of the association or concept in the consumers’ minds [59]. Additionally, only positive hedonic words, such as ‘appetizing’, ‘favourite’, ‘good’, ‘I love it’, ‘pleasant’, and ‘wonderful’, were evoked by our participants. Not surprisingly, this reinforces that the Portuguese consumer focuses on sensorial attributes of the product, which is in tune with findings that sensory appeal is one of the main determinants of food choice by Portuguese consumers [26]. ‘Appearance’ was by far the largest category of the “rice” stimulus and the main sensory attribute, followed by ‘flavour’ and ‘texture’. This means that Portuguese consumers can rely on extrinsic rice attributes (e.g., visual appearance) to assess intrinsic product attributes (e.g., texture). These findings reveal similarities between Portuguese and Asian consumers who describe a “good rice” based on the appearance attribute [35], and contrast with other consumers, namely European consumers [60], for whom the texture is a decisive attribute of quality rice [61,62,63]. This could be explained by the fact that Asian and Portuguese consumers have a vast experience in eating and cooking, enabling them to use different intrinsic and extrinsic cues when evaluating the quality of rice [64]. This similarity of behaviours of one European country and Asia is likely the result of the fusion of experiences brought about by the maritime expansion of Portugal towards South and Eastern Asia for over 500 years from the 15th century.

In fact, the second most frequent dimension for the “rice” stimulus was ‘cooking and consumption’, a dimension that aggregates categories related to the methods of preparation and ways of consuming rice. This salience could be explained by the fact that rice is part of the Portuguese cuisine, as it was considered by our participants as an everyday food (a ‘staple’ food) and an ingredient served as side dish (e.g., ‘plain rice’, ’bean rice’, ‘cabbage rice’, ‘carrot rice’, ‘*Malandro* rice’, ‘spring rice’, ‘tomato rice’) or as a main course (e.g., ‘*Cabidela* rice’, ‘chicken rice’, ‘codfish rice’, ‘duck rice’, ‘seafood rice’, ‘octopus rice’, ‘sweet rice’—dessert, ‘Valencian rice’). Additionally, participants also spontaneously evoked words related to the methods of preparing and consuming rice (e.g., ‘bake’, ‘braise’, ‘*estrugido*’ (with pre-stir fried onions in olive oil)*,* ‘grill’, ‘hot’), revealing familiarity with these culinary practices. Some of our participants seemed to be so familiar with rice’s culinary practices, that they perceived this food as ‘easy’, ‘fast’, ‘practical’, ‘variety’, and ‘versatile’ to cook (‘convenience’ dimension).

Although our participants evoked some words such as ‘carbohydrates’ and ‘calories’ (grouped in the category ‘nutritional aspects’), presumably reflecting some concern about its caloric content, other words were also found, evoking positive associations with rice’s nutritional properties, such as ‘balanced’, ‘good nutrition’, and ‘nutritious’. This shows that rice consumption is also positively related with a healthy diet, in accordance with French, Spanish, Greek, and Dutch consumers [65], as well as US consumers [66,67,68].

Even though the hedonic value dominates for the “rice” stimulus, as our participants appreciated the sensory properties of rice as it offers pleasure and evokes feelings of pleasure (experiential view) [39], the value of rice was also determined by how well it performs, namely, considering its use as a side dish or main dish, or as a source of nutritional properties or as a means to prepare a conventional meal. As a result, for our participants, the two dimensions of rice’s benefits, hedonic and utilitarian, are not mutually exclusive [69], and do not compete with each other [70], thus promoting rice consumption. Additionally, words carrying cultural or personal meaning, such as ‘childhood’, ‘family’, ‘grandmother’, and ‘party’ were also referred to by our participants. These words have already been reported in studies of traditional food conceptualization [48,71], and this symbolic view of consumption has been closely associated with ethnic and traditional food consumption [48,72,73], reinforcing the cultural/traditional identity associated with rice consumption in Portugal. By contrary, French consumers consider rice as a food from other cultures [35], emphasizing the European food cultural heterogeneity despite the geographical proximity between countries [48,74].

Considering the “rice with low GI”, consumers made significantly more associations with utilitarian values and significantly less with experiential and symbolic values. In fact, for this stimulus, the most frequent words mentioned by our consumers were ‘brown rice’, ‘healthy/health’, and ‘plain rice’, resulting in three dimensions related to the rice’s functional attributes: ‘types of rice’, ‘health’, and ‘cooking and consumption’. One may infer that eating “rice with low GI” promotes health benefits (e.g., ‘cleanse organism’, ‘slimming’, ‘good for health’, ‘good disposition’) due to their nutritional properties, as “rice with low GI” contains ‘proteins’, ‘low sugar’, ‘low carbohydrates’, or ‘fibre’. For our participants, these functional properties are essentially found in ‘brown’, ‘Basmati’, or ‘parboiled’ rice, corresponding to the rice types with the lowest GI [17,19]. Nevertheless, they also evoked ‘plain rice’ (a popular side dish usually made with white rice cooked only with water and salt and some other condiments), which may demonstrate a health concern but also a lack of knowledge because they believe that simple-cooking rice may be healthier. Additionally, in order to lose weight or for health and wellbeing reasons, participants evoked words that are related to the moderation and restriction of food consumption (e.g., ‘balanced regime’, ‘eat small amounts’, ‘few’, ‘moderation’).

Although our participants evaluated “rice with low GI” in a favourable way, in the sense that it was considered as ‘recommendable’, ‘appropriate’, ‘special’, and ‘suitable’, some also evoked negative feelings and emotions, such as ‘misinformation’, ‘uncertainty’, and ‘unfamiliarity’, and negative sensory terms, such as ‘bitter’, ‘little taste’, ‘insipid’, ‘less appealing’, ‘no-taste’, ‘tasteless’, and ‘unpleasant’. This could be a barrier to promoting consumption of “rice with low GI”, as consumers hardly compromise taste for health [72]. Similar results were obtained for functional foods, as their health/wellbeing benefits acceptance has become more conditional, particularly with respect to bad taste [75,76,77]. In the same way, “rice with low GI” may evoke contradicting perceptions, as this product is perceived to be innovative (‘development experiment’, ‘innovation’) or natural (‘additive-free’, ‘natural’, ‘organic’, ‘unprocessed’). Previous studies have shown that a product perceived to be natural is thought to be minimally processed and/or produced by traditional methods, and it also seems that naturalness is associated with desirable sensory attributes [78].

Regarding the rice consumption profile, it was found that the most frequent rice consumers (Daily cluster) were those who reported consuming all types of rice available on the market significantly more often, demonstrating that the variety of types of rice allows or facilitates such daily consumption. This contrasts with the Specialities cluster, which are the respondents with the lowest frequency of rice consumption. This cluster seems to be more selective in the type of rice, choosing the types considered exotic, which are usually more expensive. Moreover, there were significantly more respondents with higher education (28.1%) and with higher income (30.7%) in the Specialities cluster, thus confirming that the prices of this type of rice are important in the purchase decision. The importance of this factor is reinforced by the fact that Daily consumers have significantly fewer respondents with the highest income, showing in a way that rice (in general) is an affordable food. This same consumer cluster associated symbolic values to “rice” significantly more, demonstrating a certain projection of the lifestyle and cultures, and this was shown by words such as ‘brand’, ‘family’, ‘exotic’, ‘party’, ‘modernity’, and ‘sushi’, which formed this value group.

In the “rice” stimulus, the older respondents did significantly fewer associations to the ‘types of rice’ than the younger groups. This may be the result of the presence of new types of rice in the national market to which younger people are more familiar or perhaps more open to new experiences. On the other hand, for “rice with low GI”, the older age group and respondents without higher education evoked significantly more terms related to ‘low GI disconnects’. This is aligned with the findings that reveal that the level of education has significant effects on nutritional knowledge [79,80] and on healthy food perception [81].

The younger respondents referred significantly more to ‘innovation’; however, it was the older ones who showed greater interest, referring significantly more to ‘positive attitudes’. These positive associations such as ‘appropriate’, ‘suitable’, ‘safe’, ‘ideal’, and ‘recommendable’ showed that interest in the potential product is more significant for the older age group. These findings contrast with results from the United Kingdom and France, where it was found that the perception of rice differs between age groups, where rice is seen as a new food by older consumers and as a staple food by young ones [82]. The older respondents also evoked significantly more terms related to ‘naturalness’ and ‘attitudes and emotions’, which have a stronger experiential dimension. However, as the affective dimension is something acquired (experiential), the association of these categories to a hypothetical low GI product (stimulus) highlights the importance of these dimensions in food choice, especially in this age group, corroborating studies that predict a greater focus on affective and emotional issues in older consumers [81,83].

The “rice dishes” dimensions from the “rice with low GI” stimulus were significantly more often mentioned by Local consumers, which is justified by the fact that the mentioned dishes were mostly typical Portuguese dishes made with the *Carolino* rice type, the locally produced one. In this cluster there were significantly more male respondents (55.7%), while in the Specialities cluster there were significantly more female respondents (25.7%), confirming that males are less prone to new experiences and more neophobic than female consumers [84,85]. This aversion to the unknown, or low propensity for new foods shown by male respondents, can also be reinforced by the ‘negative feelings and emotions’ category of the stimulus “rice with low GI”, where they mentioned significantly more words from this group. The word “rice” evoked more associations with experiential values and the ‘sensory’ dimension for female respondents. This result suggests that women in relation to men give more importance to hedonic consumption, as found in other studies [86,87,88].

## 5. Conclusions

The FWA task showed differences between “rice” and “rice with low GI”, exploring the constructs that can shape attitudes and preferences in relation to such concepts and capturing the consumers’ perceptions and expectations in relation to the product concept.

For Portuguese consumers, rice is an everyday food and a major ingredient served as a side dish or as a main course, as they appreciate their sensory properties, offering pleasure and evoking feelings of pleasure. On the other hand, the utilitarian value dominates the consumption values of the “rice with low GI”.

“Rice with low GI” incites something new and positive for health, as normally, health benefits are connected to uncertainties regarding future health status. However, to accept this product innovation, consumers must perceive and be persuaded that health and nutritional benefits compensate for negative sensorial properties.

The relationship between values, dimensions, and categories with socio-demographic characteristics and consumption profiles allowed us to explore differences among consumers, clearly identifying four clusters of consumers: Specialities, Local, Daily, and *Agulha*. For these groups, the differences in the rice consumption patterns were clearly associated with different attitudes and perceptions towards “rice” and “rice with low GI”.

The results provide general information for the development of a consumer-oriented low GI rice product. However, future research may be oriented towards a more specific assessment of the intrinsic and extrinsic expectations of consumers in relation to this type of product.

## Figures and Tables

**Table 1:** Sociodemographic characteristics of the participants (n = 256).

Variable		Absolute Frequency	Relative Frequency
Age group (mean ± SD: 40 ± 13 years)			
	[18; 35[	90	35.2%
	[35; 55[	124	48.4%
	55+	42	16.4%
Sex			
	Female	164	64.1%
	Male	92	35.9%
Education level			
	No higher education	164	64.1%
	Higher education	92	35.9%
Net monthly per capita household income			
	≤250 €	65	25.4%
	[250–400[ €	66	25.8%
	[400–550[ €	62	24.2%
	>550 €	63	24.6%

**Table 2:** Frequencies of words and respondents (%) according to each dimension and category for the “rice” stimulus. The predominant consumption value associated with the words within each dimension is also referred.

Dimension	Category	Examples of Elicited Words	Word (%)	Respondent (%) ^1^	Predominant Consumption Value
Sensory	Appearance	appearance, colour, white, large grain, loose, size, small grain	16.3	38.3	Experiential
	Flavour	aroma, savour, smell, sweet, taste	9.8	26.2	
	Texture	*al dente*, brothy, creamy, crunchy, dry, grainy, hard, moist, parched, smooth, soft, wet, texture	4.2	10.9	
	Positive hedonics	appetizing, favourite, good, ‘I love it’, pleasant, wonderful	3.9	10.9	
Cooking and consumption	Specific foods	bacon, beans, cabbage, carrot, chicken, herbs, meat, red beans, shrimp, onion, peas, tomatoes, tuna, vegetables	8	15.6	Utilitarian
	Rice side dish	bean rice, cabbage rice, carrot rice, *Malandro* rice, side dish, spring rice, tomato rice, plain rice	7.6	21.9	
	Rice main course	*Cabidela* rice, chicken rice, codfish rice, duck rice, seafood rice, octopus rice, sweet rice (dessert), Valencian rice	5.8	14.1	
	Culinary practice	bake, braise, *estrugido*, grill, hot, oven, porridge, roast, soggy, stir-fry	2.9	7.8	
Nutrition	Staple/sustenance	basic, eating, essential, food, meal, staple, sustenance	6.8	18.4	Utilitarian
	Nutritional aspect	balanced, calories, carbohydrates, energy, good nutrition, nutritious, natural, nutritious, protein	4.3	12.5	
Types of rice *		Basmati, *Carolino*, *Agulha*, *Risotto*, brown rice, parboiled, wild, Jasmine, waxy	8.5	19.5	Utilitarian
Geography and culture		Africa, Asia, China, national, East, Thailand, exotic, chopsticks	4.8	12.9	Symbolic
Agriculture		agriculture, countryside, farming, environment, paddy field, plantation, seeds	4.1	10.2	Symbolic
Convenience		easy, fast, practical, variety, versatile	3	8.6	Utilitarian
Positive feelings and emotions		comfort, enjoyment, fun, joy, passion, pleasure, spectacular, success	3.4	8.2	Experiential
Health		health, healthy	3	8.2	Utilitarian
Family		childhood, holidays, home, family, mother, grandmother, son	2.5	5.9	Symbolic

^1^ Respondent percentages for each category. * Types of rice utilizes the nomenclature freely used by consumers, as explained in the text.

**Table 3:** Frequencies of words and respondents (%) according to each dimension and category for the “rice with low glycaemic index” stimulus. The predominant consumption value associated with the words within each dimension is also referred.

Dimension	Category	Examples of Elicited Words	Word (%)	Respondent ^1^ (%)	Predominant Consumption Values
Types of rice		brown, Basmati, wild, parboiled	19.4	41.0	Utilitarian
Nutrition	Nutritional aspects	low sugar, low carbohydrates, calories, energy, fat, fibre, nutrients, nutritive, nutrition, protein	7.0	20.3	Utilitarian
	Diet patterns	balanced regime, diet, eat small amounts, few, less, moderation	5.7	15.2	
	Low GI disconnects	gluten free, with vitamins, more vitamins, more phosphorus, without salt, fat free, high in carbohydrates, non-fat rice	4.3	11.7	
Health	Physiological	blood glucose, diabetic, health, healthy, hunger, slow absorption	18.0	38.0	Utilitarian
	Health benefits	cleanse organism, slimming, treatment, good for health, prevent, longevity, strengthening the organism, healthy life, quality of life, good disposition	2.5	6.6	
Cooking and consumption	Rice dishes	plain rice, vegetable rice, *Malandro* rice, rice soup, raisin rice	6.3	16.4	Utilitarian
	Specific foods	bean, chia, chicken, coconut water, fish, mushrooms, oat, quinoa, rice, seeds, spices, vegetables, yogurts	4.8	11.3	
	Culinary practices	boiling, confectioning, cooking, grilling, steaming, stewing	1.7	5.1	
Attitudes and emotions	Positive attitudes and emotions	advisable, alternative, appropriate, better, essential, good mood, happiness, ideal, interesting, joy, quality, recommendable, safe, satisfaction, special, suitable, wanting	4.7	12.9	Experiential
	Negative feelings and emotions	dissatisfied, doubt, expendable, misinformation, uncertainty, unfamiliarity	2.0	4.3	
Sensory	Positive	delicious, loose, multicolour, taste, tasty	3.6	10.9	Experiential
	Negative	bitter, little taste, insipid, less appealing, no-taste, tasteless, unpleasant	2.5	5.9	
Naturalness		additive-free, natural, organic, preservative-free, pure, unprocessed	4.0	10.5	Utilitarian
Supply chain		brand, chain, cost, customer, dehydrated, expensive, health and wellbeing section, instant rice, rice drink, price, tufted rice	3.0	8.2	Utilitarian
Innovation		created, develop, development experiment, innovation, laboratory, produce	2.5	5.9	Symbolic

^1^ Respondent percentages for each category.

**Table 4:** Self-reported frequency of consumption of different types of rice (meals/week) by consumption profile.

		**Rice Consumption Profile (n = 256)**			
**Types of Rice**	**Overall** **(n = 256)**	**Specialities (n = 58)**	**Local (n = 108)**	**Daily (n = 24)**	***Agulha* (n = 66)**
Rice *	4.4 (0.12)	3.6 (0.27) ^b^	4.2 (0.20) ^b^	6.8 (0.73) ^a^	4.5 (0.33) ^b^
*Agulha*	2.6 (0.08)	0.5 (0.53) ^c^	2.8 (0.15) ^b^	4.1 (0.61) ^a^	3.6 (0.31) ^a^
*Carolino*	1.9 (0.08)	1.3 (0.10) ^b^	2.8 (0.10) ^a^	4.3 (0.43) ^a^	0.3 (0.03) ^c^
Basmati	1.0 (0.05)	1.7 (0.16) ^b^	0.5 (0.04) ^c^	2.7 (0.26) ^a^	0.9 (0.09) ^c^
Parboiled	0.8 (0.05)	1.3 (0.13) ^b^	0.6 (0.07) ^c^	2.2 (0.22) ^a^	0.4 (0.57) ^c^
Brown	0.5 (0.04)	0.7 (0.07) ^b^	0.3 (0.04) ^c^	2.1 (0.24) ^a^	0.3 (0.04) ^c^
Jasmine	0.3 (0.03)	0.5 (0.06) ^b^	0.2 (0.11) ^c^	1.7 (0.23) ^a^	0.2 (0.02) ^c^
Risotto	0.2 (0.04)	0.4 (0.04) ^b^	0.2 (0.01) ^c^	0.7 (0.07) ^a^	0.2 (0.02) ^c^

^a, b, c^ Homogeneous group according to the nonparametric test of Kruskal–Wallis with 5% significance level and the pairwise comparison post hoc test. * Global rice consumption frequency as directly reported by participants. Mean (standard error).

**Table 5:** Sociodemographic characterization of the rice consumption profiles according to age group, sex, education level, and monthly household income.

		Rice Consumption Profile (n = 256)			
Sociodemographic Variables		Specialities (n = 58)	Local	High Frequency	*Agulha*
			(n = 108)	(n = 24)	(n = 66)
Age group (mean ± SD: 40 ± 13)					
	[18; 35[	18.1	43.0	12.2	26.7
	[35; 55[	25.8	41.9	8.1	24.2
	55+	22.2	43.7	4.8	29.3
Sex					
	Female	25.7 (+) ***	35.4 (−) ***	11.5 (+) ***	27.5
	Male	16.8 (−) ***	55.7 (+) ***	4.4 (−) ***	23.1
Education level					
	No higher education	19.5 (−) **	46.0 (+) **	8.4	26.1
	Higher education	28.1 (+) **	36.3 (–) **	10	25.6
Monthly household income (estimated per capita)					
	≤250 €	21.5	44.6	12.3	21.6
	[250–400[ €	18.7	45.0	12.1	24.2
	[400–550[ €	19.4	43.0	6.5	31.1
	>550 €	30.7 (+) **	37.6	4.7 (−) **	27.0

(+) or (−) indicate that the observed value is greater or less than, respectively, the expected theoretical value. ** *p* < 0.01; *** *p* < 0.001 chi-square effect per cell.

**Table 6:** Frequency (%) of elicited words according to dimensions, categories, and values in the free word association task using the ‘‘rice” stimulus according to sex, age group, education level, and rice consumption profile.

Dimension	Sex		Age Group (years)			Education Level		Rice Consumption Profile			
Category	Male	Female	[18; 35[	[35; 55[	55+	No Higher Education	Higher Education	Specialities	Local	Daily	*Agulha*
**Sensory**	30 (−) **	70 (+) **	39	46	15	68	32	16 (−) *	45	11	28
Appearance	33	67	43 (+) ***	43	14	63	37	14 (−) **	44	10	32
Flavour	32	68	30	53	17	75 (+) ***	25 (−) ***	19	49	11	21
Texture	22	78	47	41	12	63	37	16	40	16	28
Positive hedonics	20 (−) **	80 (+) **	33	53	14	77	23	20	43	14	23
**Cooking and consumption**	45 (+) **	55 (−) **	34	55 (+) ***	11 (−) ***	57 (−) ***	43 (+) ***	22	48	8	22
Specific foods	52 (+) **	48 (−) **	31	67 (+) ***	2 (−) ***	62	38	16	52	16	16
Rice side dish	43	57	33	47	20	50 (−) ***	50 (+) ***	26	45	7	22
Rice main course	45	55	36	50	14	61	39	30	42	5	23
Culinary practice	32	68	41	55	4	50	50	19	49	11	21
**Nutrition**	34	66	34	42	24 (+) ***	67	33	26	42	10	22
Staple/sustenance	27	73	29	46	25	67	33	29	36	8	27
Nutritional aspect	45	55	43	36	21	67	33	21	52	12	15
**Types of rice**	25 (−) **	75 (+) **	45	53	2 (−) ***	44 (−) ***	56 (+) ***	34 (+) *	31 (−) *	7	28
**Positive feelings and emotions**	31	69	8 (−) ***	57	35 (+) ***	85 (+) ***	15 (−) ***	15	19 (−) **	4	62 (+) **
**Geography and culture**	53 (+) **	47 (−) **	36	56	8	67	33	19	47	12	22
**Agriculture**	45	55	32	32	36 (+) ***	65	35	42 (+) *	29	32	26
**Convenience**	30	70	39	30	31 (+) ***	87 (+) ***	13 (−) ***	22	39	26 (+) *	13
**Health**	35	65	13 (−) ***	35	52 (+) ***	96 (+) ***	4 (−) ***	22	43	5	30
**Family**	18 (−) **	82 (+) **	23	64	13	64	36	36	32	5	27
**Consumption values**											
Utilitarian	39	61	36	48	16	61	39	25	43	9	23
Experiential	29 (−) *	71 (+) *	35	48	16	69	31	16 (−) *	43	11	30 (+) *
Symbolic	42	58	32	48	20	66	34	32 (+) *	38	7	23

Effect of the chi-square per cell. (+) or (−) indicate that the observed value is higher or lower than the expected theoretical value: * *p* < 0.05; ** *p* < 0.01; *** *p* < 0.001.

**Table 7:** Frequency (%) of elicited words according to dimensions, categories, and consumption values in the free word association task using the ‘‘rice with low glycaemic index” stimulus according to sex, age group, education level, and rice consumption profile.

Dimension	Sex		Age Group (Years)			Education		Rice Consumption Profile			
Category	Male	Female	[18; 35[	[35; 55[	55+	No Higher Education	Higher Education	Specialities	Local	Daily	*Agulha*
**Type of rice**	19 (−) ***	81 (+) ***	40	49	11 (−) ***	54 (−) **	46 (+) **	34 (+) *	34 (−) *	8	24
**Nutrition**	33	67	34	48	18	90	42	24	41	10	25
Nutritional aspect	35	65	50 (+) ***	43	7 (−) ***	56	44	28	35	9	28
Diet patterns	33	47	30	48	22	70	30	18	48	11	23
Low GI disconnects	30	70	15 (−) ***	58	27	88 (+) **	12 (−) **	27	39	9	24
**Health**	41	59	33	50	17	66	34	16 (−) *	45	12	27
Physiological	40	60	32	52	16	65	35	16	45	10	29
Health benefits	11	10	37	42	21	74	26	16	47	21	16
**Cooking and consumption**	39	61	21 (−) ***	68 (+) ***	9	76 (+) *	24 (−) *	26	50	7	17 (−) *
Rice dishes	31	69	27	58	15	79 (+) **	21 (−) **	21	56 (+) *	6	17
Specific foods	46	54	16 (−) ***	81 (+) ***	3	76	24	30	43	8	19
Culinary practices	46	54	15	70	15	62	38	31	46	8	15
**Attitudes and emotions**	50 (+) ***	50 (−) ***	24	42	34 (+) ***	68	32	18	46	6	30
Positive attitudes	44	56	22	33 (−) ***	45 (+) ***	75	25	19	39	8	33
Negative feelings and emotions	69 (+) ***	31 (−) ***	31	56	13	56	44	13	63 (+) *	6	19
**Sensory**	40	60	42	51	7 (−) *	60	40	7 (−) *	49	12	33
Positive	39	61	32	57	11	61	39	16 (−) *	45	10	29
Negative	40	60	60 (+) ***	33	7 (−) ***	60	40	7	53	20	20
**Naturalness**	35	65	32	39	29 (+) ***	65	35	16	42	3	39
**Supply chain**	26	74	35	39	26	65	35	22	43	13	22
**Innovation**	68 (+) ***	32 (−) ***	74 (+) ***	21 (−) ***	5	32 (−) **	68 (+) **	11	42	16	32
**Consumption values**											
Utilitarian	33	67	35	50	15	64	36	24 (+) *	41 (−) *	10	25
Experiential	42	58	33	46	21	65	35	13 (−) *	50 (+) *	7	30
Symbolic	44	56	43	44	13	56	44	6	50	19	25

Effect of the chi-square per cell. (+) or (–) indicate that the observed value is higher or lower, respectively, than the expected theoretical value: * *p* < 0.05; ** *p* < 0.01; *** *p* < 0.001.

## Data Availability

The data presented in this study are available upon request from the corresponding author.

## References

[1] FAO Food Balance Sheets. http://faostat.fao.org/.

[2] Dias A.S., Dias L.S. (2018). A Data Set of Portuguese Traditional Recipes Based on Published Cookery Books. Data.

[3] Lopes C., Torres D., Oliveira A., Severo M., Alarcão V., Guiomar S., Mota J., Teixeira P., Rodrigues S., Lobato L. (2017). Inquérito Alimentar Nacional e de Atividade Física, IAN-AF 2015-2016: Relatório de Resultados.

[4] Arroz C.d.A.-O.I.d. Dados da Fileira do Arroz em Portugal. http://app.parlamento.pt/webutils/docs/doc.pdf.

[5] Seah J.Y.H., Koh W.P., Yuan J.M., Van Dam R.M. (2018). Rice intake and risk of type 2 diabetes: The Singapore Chinese Health Study. Eur. J. Nutr..

[6] Golozar A., Khalili D., Etemadi A., Poustchi H., Fazeltabar A., Hosseini F., Kamangar F., Khoshnia M., Islami F., Hadaegh F. (2017). White rice intake and incidence of type-2 diabetes: Analysis of two prospective cohort studies from Iran. BMC Public Health.

[7] Saneei P., Larijani B., Esmaillzadeh A. (2017). Rice consumption, incidence of chronic diseases and risk of mortality: Meta-analysis of cohort studies. Public Health Nutr..

[8] Mohan V., Anjana R.M., Gayathri R., Ramya Bai M., Lakshmipriya N., Ruchi V., Balasubramaniyam K.K., Jakir M.M., Shobana S., Unnikrishnan R. (2016). Glycemic Index of a Novel High-Fiber White Rice Variety Developed in India—A Randomized Control Trial Study. Diabetes Technol. Ther..

[9] Hu E.A., Pan A., Malik V., Sun Q. (2012). White rice consumption and risk of type 2 diabetes: Meta-analysis and systematic review. BMJ.

[10] Neal B. (2012). White rice and risk of type 2 diabetes. BMJ.

[11] Jenkins D.J., Wolever T.M., Taylor R.H., Barker H., Fielden H., Baldwin J.M., Bowling A.C., Newman H.C., Jenkins A.L., Goff D.V. (1981). Glycemic index of foods: A physiological basis for carbohydrate exchange. Am. J. Clin. Nutr..

[12] Brand-Miller J., Foster-Powell K. (2006). The New Glucose Revolution: The Authoritative Guide to the Glycemic Index—The Dietary Solution for Lifelong Health.

[13] Augustin L.S., Kendall C.W., Jenkins D.J., Willett W.C., Astrup A., Barclay A.W., Björck I., Brand-Miller J.C., Brighenti F., Buyken A.E. (2015). Glycemic index, glycemic load and glycemic response: An International Scientific Consensus Summit from the International Carbohydrate Quality Consortium (ICQC). Nutr. Metab. Cardiovasc. Dis..

[14] Henry C.J., Quek R.Y.C., Kaur B., Shyam S., Singh H.K.G. (2021). A glycaemic index compendium of non-western foods. Nutr. Diabetes.

[15] Wee M.S.M., Henry C.J. (2020). Reducing the glycemic impact of carbohydrates on foods and meals: Strategies for the food industry and consumers with special focus on Asia. Compr. Rev. Food Sci. Food Saf..

[16] Teixeira J.M.L.C.C. (2013). Estudos de Índice Glicémico de Variedades de Arroz. Master’s Thesis.

[17] Foster-Powell K., Holt S.H., Brand-Miller J.C. (2002). International table of glycemic index and glycemic load values: 2002. Am. J. Clin. Nutr..

[18] Kabir E., Hossain M., Hossain M., Ray S., Bhuiyan M.J. (2021). Glycemic Index Values of Rice Varieties that are Commonly Available in Markets in Bangladesh. J. Gizi Dan Pangan.

[19] Atkinson F.S., Foster-Powell K., Brand-Miller J.C. (2008). International tables of glycemic index and glycemic load values: 2008. Diabetes Care.

[20] Kumar A., Lal M.K., Nayak S., Sahoo U., Behera A., Bagchi T.B., Parameswaran C., Swain P., Sharma S. (2022). Effect of parboiling on starch digestibility and mineral bioavailability in rice (*Oryza sativa* L.). LWT.

[21] Graça P., Gregório M.J., Freitas M.G. (2020). A Decade of Food and Nutrition Policy in Portugal (2010–2020). Port. J. Public Health.

[22] DGS (2017). A Saúde dos Portugueses 2016.

[23] Barreto M., Kislaya I., Gaio V., Rodrigues A.P., Santos A.J., Namorado S., Antunes L., Gil A.P., Boavida J.M., Ribeiro R.T. (2018). Prevalence, awareness, treatment and control of diabetes in Portugal: Results from the first National Health examination Survey (INSEF 2015). Diabetes Res. Clin. Pract..

[24] Rito A., Mendes S., Baleia J., Gregório M.J. Childhood Obesity Surveillance Initiative. COSI Portugal 2019. https://www.ceidss.com/wp-content/uploads/2021/10/COSI_Portugal_2019_out2021.pdf.

[25] Webster A.D., Soli K. (2018). Is Everyone Really on a Low-Carbohydrate Diet? Consumer Perceptions of Carbohydrates and Sugars. Cereal Foods World.

[26] Cunha L.M., Cabral D., Moura A.P., De Almeida M.D.V. (2018). Application of the Food Choice Questionnaire across cultures: Systematic review of cross-cultural and single country studies. Food Qual. Prefer..

[27] Ares G., De Saldamando L., Giménez A., Claret A., Cunha L.M., Guerrero L., De Moura A.P., Oliveira D.C.R., Symoneaux R., Deliza R. (2015). Consumers’ associations with wellbeing in a food-related context: A cross-cultural study. Food Qual. Prefer..

[28] Jaeger S.R., Hort J., Porcherot C., Ares G., Pecore S., MacFie H.J.H. (2017). Future directions in sensory and consumer science: Four perspectives and audience voting. Food Qual. Prefer..

[29] Köster E.P. (2009). Diversity in the determinants of food choice: A psychological perspective. Food Qual. Prefer..

[30] Belk R., Fischer E., Kozinets R.V. (2012). Qualitative Consumer and Marketing Research.

[31] Donoghue S. (2000). Projective techniques in consumer research. J. Fam. Ecol. Consum. Sci..

[32] Smith S.M., Albaum G.S. (2010). An Introduction to Marketing Research.

[33] Roininen K., Arvola A., Lähteenmäki L. (2006). Exploring consumers’ perceptions of local food with two different qualitative techniques: Laddering and word association. Food Qual. Prefer..

[34] Rozin P., Kurzer N., Cohen A.B. (2002). Free associations to “food:” the effects of gender, generation, and culture. J. Res. Personal..

[35] Son J.-S., Do V.B., Kim K.-O., Cho M.S., Suwonsichon T., Valentin D. (2014). Understanding the effect of culture on food representations using word associations: The case of “rice” and “good rice”. Food Qual. Prefer..

[36] Lanseng E. (2011). A Typology of Consumption Value: Teasing Out the Unique Properties of Utilitarian, Symbolic, Experiential, and Aesthetic Consumption Qualities. Proceedings of the European Advances in Consumer Research.

[37] Charters S. (2006). Wine and Society: The Social and Cultural Context of a Drink.

[38] Werle C.O.C., Trendel O., Ardito G. (2013). Unhealthy food is not tastier for everybody: The “healthy=tasty” French intuition. Food Qual. Prefer..

[39] Holbrook M.B., Hirschman E.C. (1982). The Experiential Aspects of Consumption: Consumer Fantasies, Feelings, and Fun. J. Consum. Res..

[40] Rintamäki T., Kanto A., Kuusela H., Spence M.T. (2006). Decomposing the value of department store shopping into utilitarian, hedonic and social dimensions. Int. J. Retail. Distrib. Manag..

[41] Letarte A., DubÉ L., Troche V. (1997). Similarities and Differences in Affective and Cognitive Origins of Food Likings and Dislikes. Appetite.

[42] Aaker D.A., Kumar V., Day G.S. (2008). Marketing Research.

[43] Welch A.A., Caballero B. (2013). Dietary intake measurement: Methodology. Encyclopedia of Human Nutrition.

[44] Krippendorff K. (2018). Content Analysis: An Introduction to its Methodology.

[45] Modell S. (2005). Triangulation between case study and survey methods in management accounting research: An assessment of validity implications. Manag. Account. Res..

[46] Anderson R.B.W., Brislin R.W. (1976). Translation: Applications and Research.

[47] De Andrade J.C., De Aguiar Sobral L., Ares G., Deliza R. (2016). Understanding consumers’ perception of lamb meat using free word association. Meat Sci..

[48] Guerrero L., Claret A., Verbeke W., Enderli G., Zakowska-Biemans S., Vanhonacker F., Issanchou S., Sajdakowska M., Granli B.S., Scalvedi L. (2010). Perception of traditional food products in six European regions using free word association. Food Qual. Prefer..

[49] Schmitt N. (1998). Quantifying word association responses: What is native-like?. System.

[50] Sánchez-Fernández R., Iniesta-Bonillo M.Á. (2007). The concept of perceived value: A systematic review of the research. Mark. Theory.

[51] Sheth J.N., Newman B.I., Gross B.L. (1991). Why we buy what we buy: A theory of consumption values. J. Bus. Res..

[52] Holt D.B. (1995). How Consumers Consume: A Typology of Consumption Practices. J. Consum. Res..

[53] Hirschman E.C., Holbrook M.B. (1982). Hedonic Consumption: Emerging Concepts, Methods and Propositions. J. Mark..

[54] Symoneaux R., Galmarini M.V., Mehinagic E. (2012). Comment analysis of consumer’s likes and dislikes as an alternative tool to preference mapping. A case study on apples. Food Qual. Prefer..

[55] Sharpe D. (2015). Chi-Square Test is Statistically Significant: Now What?. Pract. Assess. Res. Eval..

[56] Agresti A. (2013). Categorical Data Analysis.

[57] Bortfeld H., Leon S.D., Bloom J.E., Schober M.F., Brennan S.E. (2001). Disfluency Rates in Conversation: Effects of Age, Relationship, Topic, Role, and Gender. Lang. Speech.

[58] Merlo S., Mansur L.L. (2004). Descriptive discourse: Topic familiarity and disfluencies. J. Commun. Disord..

[59] Guerrero L., Colomer Y., Guàrdia M.D., Xicola J., Clotet R. (2000). Consumer attitude towards store brands. Food Qual. Prefer..

[60] D’Hauteville F., Aurier P., Sirieix L. A sensory approach to consumer’s preferences for rice. First results of a European survey. Proceedings of the International Symposium in Rice Quality.

[61] Okabe M. (1979). Texture measurement of cooked rice and its relationship to the eating quality. J. Texture Stud..

[62] Juliano B.O. (1993). Rice in Human Nutrition.

[63] Suwannaporn P., Linnemann A. (2008). Rice-eating quality among consumers in different rice grain preference countries. J. Sens. Stud..

[64] Grunert K.G. (2005). Food quality and safety: Consumer perception and demand. Eur. Rev. Agric. Econ..

[65] D’Hauteville F. European consumers’ rice perception. Proceedings of the Prospects for Rice Consumption in Europe Symposium.

[66] Batres-Marquez S.P., Jensen H.H., Upton J. (2009). Rice Consumption in the United States: Recent Evidence from Food Consumption Surveys. J. Am. Diet. Assoc..

[67] Nicklas T., O’Neil C., Fulgoni V. (2014). Rice Consumption Is Associated with Better Nutrient Intake and Diet Quality in Adults: National Health and Nutrition Examination Survey (NHANES) 2005–2010. Food Nutr. Sci..

[68] Kennedy E., Luo H. (2015). Association between rice consumption and selected indicators of dietary and nutritional status using National Health and Nutrition Examination Survey 2007–2008. Ecol. Food Nutr..

[69] Kalogeras N., Odekerken-Schröder G., Pennings J.M.E., Gunnlaugsdόttir H., Holm F., Leino O., Luteijn J.M., Magnússon S.H., Pohjola M.V., Tijhuis M.J. (2012). State of the art in benefit–risk analysis: Economics and Marketing-Finance. Food Chem. Toxicol..

[70] Ueland Ø., Gunnlaugsdottir H., Holm F., Kalogeras N., Leino O., Luteijn J.M., Magnússon S.H., Odekerken G., Pohjola M.V., Tijhuis M.J. (2012). State of the art in benefit-risk analysis: Consumer perception. Food Chem. Toxicol..

[71] Wang O., Gellynck X., Verbeke W. (2016). Perceptions of Chinese traditional food and European food among Chinese consumers. Br. Food J..

[72] Verbeke W., Poquiviqui López G. (2005). Ethnic food attitudes and behaviour among Belgians and Hispanics living in Belgium. Br. Food J..

[73] Vanhonacker F., Lengard V., Hersleth M., Verbeke W. (2010). Profiling European traditional food consumers. Br. Food J..

[74] Askegaard S., Madsen T.K. (1998). The local and the global: Exploring traits of homogeneity and heterogeneity in European food cultures. Int. Bus. Rev..

[75] Verbeke W. (2006). Functional foods: Consumer willingness to compromise on taste for health?. Food Qual. Prefer..

[76] Moons I., Barbarossa C., De Pelsmacker P. (2018). The Determinants of the Adoption Intention of Eco-friendly Functional Food in Different Market Segments. Ecol. Econ..

[77] Temesi Á., Bacsó Á., Grunert K.G., Lakner Z. (2019). Perceived Correspondence of Health Effects as a New Determinant Influencing Purchase Intention for Functional Food. Nutrients.

[78] Siegrist M. (2008). Factors influencing public acceptance of innovative food technologies and products. Trends Food Sci. Technol..

[79] Delley M., Brunner T.A. (2019). Breakfast eating patterns and drivers of a healthy breakfast composition. Appetite.

[80] Oliver T.L., McKeever A., Shenkman R., Diewald L. (2020). Barriers to Healthy Eating in a Community That Relies on an Emergency Food Pantry. J. Nutr. Educ. Behav..

[81] Bartkiene E., Steibliene V., Adomaitiene V., Juodeikiene G., Cernauskas D., Lele V., Klupsaite D., Zadeike D., Jarutiene L., Guiné R.P.F. (2019). Factors Affecting Consumer Food Preferences: Food Taste and Depression-Based Evoked Emotional Expressions with the Use of Face Reading Technology. BioMed Res. Int..

[82] D’Hauteville F. A synthetic analysis of six reports: France, Greece, Italy, Portugal, Spain, United Kingdom. Proceedings of the Séminaire de Montpellier.

[83] Peters E., Hess T.M., Västfjäll D., Auman C. (2007). Adult Age Differences in Dual Information Processes: Implications for the Role of Affective and Deliberative Processes in Older Adults’ Decision Making. Perspect. Psychol. Sci..

[84] Muhammad R., Abdullah K.M., Zahari M.S.M., Sharif M.S.M. (2015). Revealing the Scenario of Food Neophobia among Higher Learning Institution Students from Klang Valley, Malaysia. Procedia-Soc. Behav. Sci..

[85] Tuorila H., Lähteenmäki L., Pohjalainen L., Lotti L. (2001). Food neophobia among the Finns and related responses to familiar and unfamiliar foods. Food Qual. Prefer..

[86] Tifferet S., Herstein R. (2012). Gender differences in brand commitment, impulse buying, and hedonic consumption. J. Prod. Brand Manag..

[87] Buczek L., Migliaccio J., Petrovich G.D. (2020). Hedonic Eating: Sex Differences and Characterization of Orexin Activation and Signaling. Neuroscience.

[88] Roininen K., LÄHteenmÄKi L., Tuorila H. (1999). Quantification of Consumer Attitudes to Health and Hedonic Characteristics of Foods. Appetite.

